# Immunotherapy in Xeroderma Pigmentosum: a case of advanced cutaneous squamous cell carcinoma treated with cemiplimab and a literature review

**DOI:** 10.18632/oncotarget.27966

**Published:** 2021-05-25

**Authors:** Marco Rubatto, Martina Merli, Gianluca Avallone, Andrea Agostini, Luca Mastorino, Virginia Caliendo, Amelia Barcellini, Viviana Vitolo, Francesca Valvo, Maria Teresa Fierro, Simone Ribero, Pietro Quaglino

**Affiliations:** ^1^Department of Medical Sciences, Section of Dermatology, University of Turin, Turin, Italy; ^2^National Center of Oncological Hadrontherapy (Fondazione CNAO), Pavia, Italy; ^*^Co-first authors; ^#^Co-first senior authors

**Keywords:** xeroderma pigmentosum, immunotherapy, cemiplimab, hadrontherapy, advanced squamous cell carcinoma

## Abstract

Xeroderma Pigmentosum (XP) is a rare genetic disorder with a poor prognosis due to high photosensitivity in affected patients.

Herein, we describe the first case of the use of cemiplimab in a patient with XP, a 19-year-old girl presented with locally advanced squamous cell carcinoma of the right periorbital and nasal region. This treatment has been undertaken after a cycle of proton beam radiotherapy.

Besides, it is reported a description of the few cases in the literature describing the effectiveness of immunotherapy on skin cancers in XP-patients.

This case is in line with those reported, underlining how anti-PD1 monoclonal antibodies may be a promising treatment in this genodermatosis.

## INTRODUCTION

Xeroderma Pigmentosum (XP) is a rare genodermatosis caused by an autosomal recessive genetic defect of the nucleotide excision repair (NER) pathway. This malfunction leads to a dramatic susceptibility to UV-radiation-induced damage as the DNA repair system fails. The main clinical manifestations affect the skin and include diffuse photodamage and skin cancers presenting at an early age [1]. A 10,000-fold increased risk of developing non-melanoma skin cancers (NMSCs) compared to the general population and a 2,000-fold risk of melanoma before the age of 20 have been reported [2]. XP-patients often suffer from various ophthalmologic and neurological disorders due to UV-radiation exposure and, perhaps, to increased oxidative damage [1].

This disorder has an estimated prevalence of 2–3 per million in Western Europe, 1 per million in the United States, and 45 per million in Japan [3]. Due to its rarity, there is not an established standard of care. To date, effective management is represented by careful protection against natural and artificial UV exposure, and early detection of skin cancers, which allows their treatment with surgery and topical drugs.

The indication of radiotherapy in advanced tumours is still debated because of the well-known radiosensitivity in these patients. Conversely, immunotherapy with anti PD1/PD-L1 antibodies represents a relevant strategy in advanced NMSCs also in the context of syndromic pathologies as XP.

Herein, we report the first case of Cemiplimab used in association with hadrontherapy in a young XP-patient with an advanced cutaneous squamous cell carcinoma (cSCC) of the right orbital area.

## CASE REPORT

A 19-year-old girl affected by XP had come to our attention for a suspected advanced cSCC.

She was born in Sicily and, initially, she was followed by the dermatological centers in that region. No other family member was affected by the same pathological condition and her parents were not consanguineous. In her medical history, ophthalmic and neurological affections were not reported. Regarding skin involvement, from 5 to 13 years of age, she underwent repeated surgical excisions for a total of 24 neoformations which, on histological examination, were found to be basal cell carcinomas (BCCs) and cSCCs. The last resection was at the age of 14 due to a cSCC of the right lower eyelid. At 18 years, she received vismodegib treatment for an advanced BCC developing alopecia as an adverse event.

We firstly visited the patient in December 2018. On skin examination, a large hard swelling of the right superior eyelid extending to the nasal pyramid has been observed. An incisional biopsy was performed, resulting in a moderately-differentiated SCC. A CT scan was carried out to exclude secondary localizations. The MRI of the facial massif showed an infiltrating lesion of 62 × 47 mm. It extended from the right upper eyelid region affecting the orbital cavity’s medial wall and dislocating the eyeball from which it appeared inseparable. It jutted medially into the nasal cavities infiltrating the nasal septum and, below that, it crossed the floor of the orbit projecting into the ipsilateral maxillary sinus.

**Figure 1:**
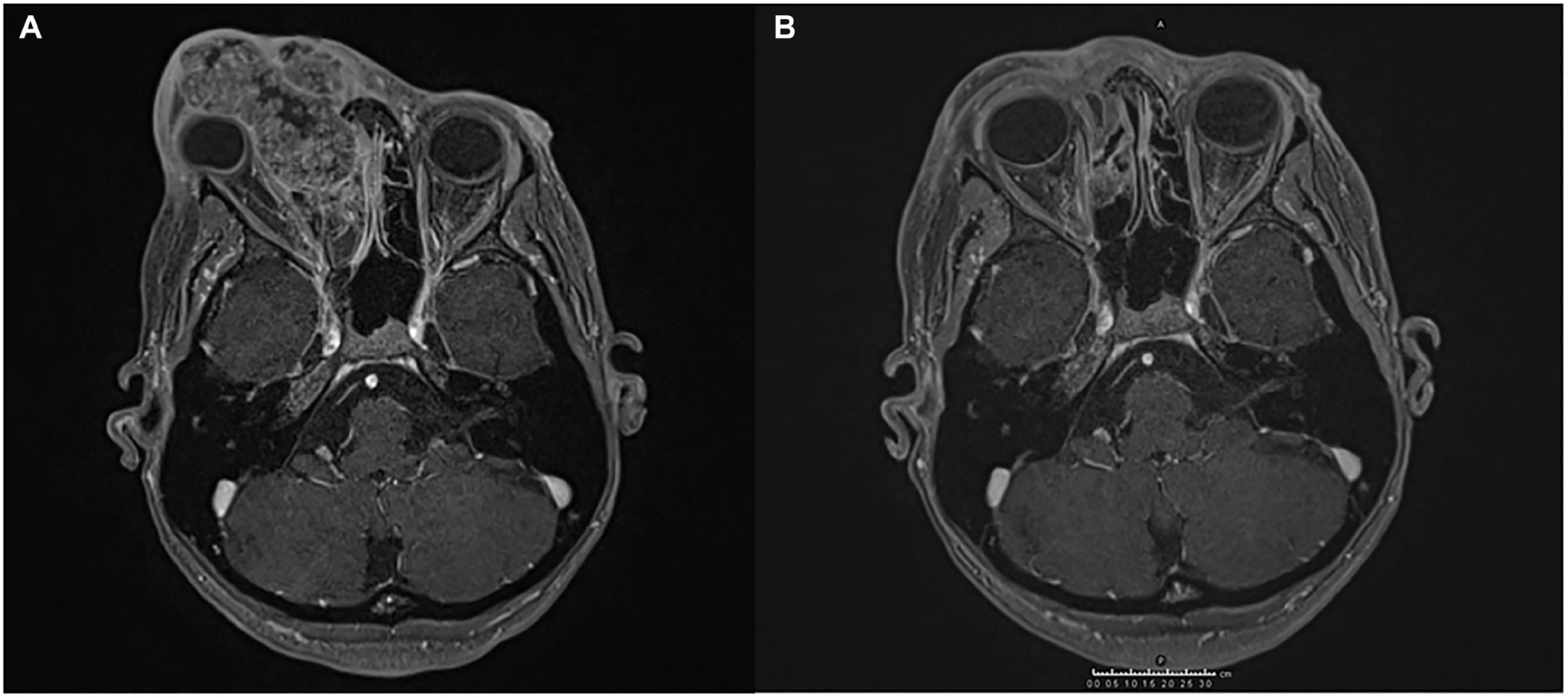
Magnetic resonance imaging (MRI) showing the response of SCC to the treatment administered. (**A**) An infiltrating lesion engages the medial wall of the right orbital cavity dislocating the eyeball, and invades the right nasal cavity and the nasal septum. (**B**) The lesion is significantly reduced after hadrontherapy.

The interdisciplinary consultation did not indicate surgical treatment, therefore the anti PD-1 cemiplimab was requested in the nominate use programme and it was decided to add radiotherapy. Pending authorization of the drug, the patient was qualified for proton beam radiotherapy (PBR), which was performed with a total dose of 59.4 Gy in 33 fractions from January to March 2019. The treatment was well tolerated and, in consideration of the remarkable clinical and radiological response, a close clinical and radiological follow-up was established. Subsequent MRI showed stable disease with only a few spots of enhancement in the radio-treated area. (Figure 1).

Meanwhile, several skin lesions were surgically removed in the following months, specifically seven BCCs and one cSCC of the face and upper limbs.

**Figure 2:**
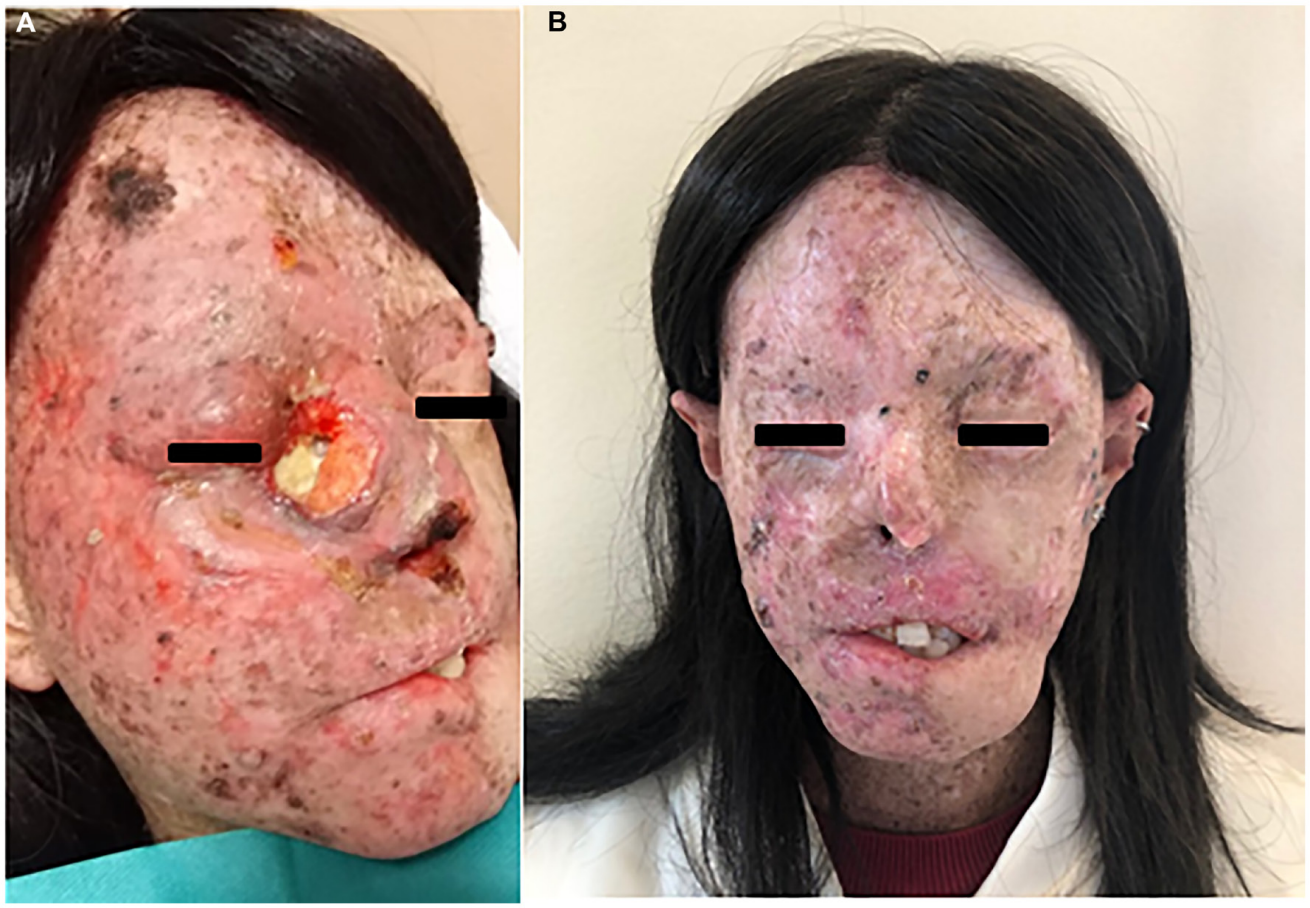
(**A**) SCC of the right eyelid extending to the nasal pyramid in a XP-patient before starting hadrontherapy. The patient after five infusions of Cemiplimab (**B**).

In June 2020, the follow-up MRI showed a laterocervical lymph node progression with a pathological submandibular lesion of 2 × 2 cm. For this reason, the treatment with cemiplimab 350 mg was initiated with administration every 3 weeks. After the first 2 infusions episodes of diarrhoea CTCAE Grade 1 were reported for which it was not necessary to stop the therapy. After 7 infusions the pathology was clinically and radiologically stable and the treatment was well tolerated without any of the other known adverse events. In addition, the patient did not have to undergo any further surgical excision as some of the lesions, especially on the face and neck, with clinical and dermoscopic parameters of actinic keratoses and BCCs had regressed. (Figure 2).

## DISCUSSION

First in 2014, with the approval of pembrolizumab for the treatment of unresectable or metastatic melanoma with disease progression following ipilimumab [4], and then in 2018, with consent to the use of cemiplimab for advanced cSCC [5], the immunotherapeutic approach has begun to take hold in non-melanoma skin cancer treatment. Immune checkpoint inhibitors (ICIs) interfere with the escape mechanisms used by tumoral cells to evade immunosurveillance. Treatment of XP-associated advanced skin tumours with ICIs has been previously described, and, in order to shed light on outcome of their administration, we gathered all the cases reported in the available literature on Pubmed so far (Table 1) [6–12]. To the best of our knowledge, our case is the first in which cemiplimab was used. Specifically, it is a fully human IgG4 antibody against PD-1 (programmed death receptor-1) that blocks its interaction with PD-L1 and 2 (programmed death ligands 1 and 2) [5].

**Table 1:** Anti-PD1 drugs used in patients with XP and their outcomes

Author^a^	Age	Sex	Group^b^	Treated tumor	Localization	Treatment	Outcome	Non-target lesions	AEs
Hauschild *et al*. [6]	51 y	M	XPE	Metastatic melanoma (pT1b, N2a, M1c)	Left cheek with lung, lymph node and right infraorbital metastases	Pembrolizumab 2 mg/kg every 3 weeks	A 90% decrease of the largest lung metastasis and a complete disappearance of the others after 3 months administration	Disappearance of almost all NMSCs and AK in the first 3 months of therapy	Reddish swelling of the skin affected by tumor lesions, mild itching
Chambon *et al*. [7]	6 y	F	XPC	Sarcomatoid carcinoma	Scalp with bone lysis, meningeal contact and superior sagittal sinus compression	At first Nivolumab 3 mg/kg every 2 weeks and the monthly (with Cetuximab for 4 cycles)	A 65% tumor volume reduction after 6 infusions with 2-weekly administration	Appearance of a SCC and two melanomas of the scalp, and others skin tumors on the skin, lip and tongue	-
Deinlein *et al*. [8]	48 y	F	-	Metastatic SCC	Left tight with lymph node metastases	Pembrolizumab 2 mg/kg every 3 weeks	Partial response with regression of all metastases after 3 cycles	-	-
Salomon *et al*. [9]	17 y	M	XPC	Metastatic melanoma (pT4b, N0, M1c)	Scalp with hepatic and lung metastases	Pembrolizumab 2 mg/kg every 3 weeks	Partial response with regression of all metastases after 4 cycles	Regression of many pre-existing NMSCs and AK after 4 cycles	Vitiligo-like depigmentation
Ameri *et al*. [10]	18 y	F	XPC	Unresectable SCC	Limbus of right eye	Pembrolizumab 2 mg/kg every 3 weeks	Complete regression after 8 months of therapy	Not response of BCCs on the face surgically removed	-
Ameri *et al*. [10]	19 y	M	XPE	Unresectable SCC	Right nasal cavity and orbit	Pembrolizumab 2 mg/kg every 3 weeks	Partial radiographic regression	-	-
Ameri *et al*. [10]	20 y	F	XPV	(i) Metastatic melanoma; (ii) unresectable SCC	(i) Unknown primary origin; (ii) maxillary sinus	(i) Ipilimumab 10 mg/kg every 3 weeks; (ii) Pembrolizumab 140 mg once a month	(i) Remarkable response; (ii) well response for 31 months until radiographic progression	Development of one BCC on right eyebrow treated with Mohs surgery	-
Steineck *et al*. [11]	7 y	F	XPC	Metastatic SCC	Right side of the face with, at first, the involvement of the right sphenoid bone, the cavernous sinus and the right carotid artery, and then the extension to surrounding tissues with lymph node metastases and leptomeningeal spread	Pembrolizumab 2 mg/kg every 3 weeks	A considerable decrease in tumor bulk and the resolution of leptomeningeal disease after five cycles; a long-term sustained stable disease	-	-
Momen *et al*. [12]	32 y	M	XPC	Cutaneous angiosarcoma	Left supraorbital area with submandibular, lung, pleural, mediastinal, pericardial, liver, and bone metastases	Pembrolizumab 200 mg every 3 weeks	Resolution of the lung and bone disease, almost complete resolution of the cardiac and pericardial involvement, and significant reduction in the liver metastases after 4 cycles	-	-

Abbreviations: SCC: squamous cell carcinoma; NMSCs: non melanoma skin cancers; AK: actinic keratoses. ^a^Superscript numbers are for the references cited in this table. ^b^There are eight different complementation groups depending on the mutated protein of NER system (XP-A through G and V) [12].

In the skin of XP-patients the lack of a valid system for repairing the UV-induced damage of DNA leads to the development of skin tumours with a likelihood of higher mutational burden (TMB) [6, 11, 12]. This accumulation of somatic mutations results in the expression of neo-antigens that elicit successful T-cell-dependent immune responses against tumor cells. For this reason, these tumors have a higher likelihood of immunotherapy response [13].

Hauschild *et al.* [6] were the first to report the use of an anti PD-1drug in a patient with XP, who was affected by metastatic melanoma. The administration of pembrolizumab allowed a notable regression of the metastases after 3 months of treatment. Besides, it was also reported the disappearance of almost all NMSCs and actinic keratoses, especially on the patient’s face. Salomon and colleagues [9] described a similar case of good response of metastatic melanoma after 4 cycles of therapy with pembrolizumab, and they also reported the regression of many pre-existing skin lesions after the first few administrations of the drug. The effectiveness of the treatment on non-target lesions represents further success on patients who have already undergone multiple exeresis, since avoiding further surgical procedures already represents an improvement in quality of life.

The patient described by Deinlein *et al.* [8] is the first reported on the efficacy of pembrolizumab on XP-associated metastatic cSCC and, similarly to the cases mentioned above, rapid response to treatment was observed after only three cycles. In another case of metastatic cSCC the patient received pembrolizumab for 24 months, with stable imaging findings for more than 18 months. This represents a good example of how ICIs can allow long-term sustained stable disease without clinically significant adverse effects [11]. To date, there are few options for alternative therapies in patients affected by XP who develop an advanced skin tumour. In this regard, immunotherapy could increase progression-free survival with minimal adverse effects, even for long periods of treatment. Among the cases described, no adverse events with a grade >2 were reported that required discontinuation of therapy [6, 9].

The immunotherapy was also effective in two cases where traditional chemotherapy did not work, specifically nivolumab against a sarcomatoid carcinoma of the scalp [7] and pembrolizumab for a cutaneous angiosarcoma of the face [12].

As reported in our case, cemiplimab may be an additional option available for these patients. We used it on a cSCC progressed after treatment of hadrontherapy that, unlike traditional radiotherapy, involves the use of proton beam. PBR has a better dose conformity compared to even the most modern photon beam radiotherapy techniques and the choice of PBR approach is coherent with the efforts to limit the dose to healthy and radiosensitive surrounding tissue which means reducing the risk of late post-actinic morbidities [14]. In our case, the anti PD-1 drug allowed to stabilize the disease with a good tolerance by the patient. In addition, the patient did not have to undergo other surgical procedures as no other skin cancers appeared and some lesions even regressed on her face.

Ameri *et al*. [10] suggested the potential of ICIs in XP-patients would extend from changing the prognostic landscape in advanced malignancies to prevention of development of others skin cancers. This would be a remarkable change for a disfiguring disease as XP that has an average survival of 32 years [2]. On the other hand, it is also to consider the potential limitation of PD-1 inhibitors for the treatment of the involvement of immune-privileged sites, such as the eye which is often affected in these patients [11].

In conclusion, XP is a good model for the study of effectiveness of the immunotherapy on skin tumours due to its high predisposition to develop them and to immunogenicity based on high mutational burden in the skin of affected patients. However, given the limited data on the disease, further studies are needed to identify the correct dosage and infusion rate of anti PD-1 drugs.

For the time being, experience and knowledge available to date on non-syndromic tumors shed light and provide important information also for rare diseases such as XP, for which there are currently no clinical trials.
