# Addendum: Immunotherapy in Xeroderma Pigmentosum: a case of advanced cutaneous squamous cell carcinoma treated with cemiplimab and a literature review

**DOI:** 10.18632/oncotarget.28265

**Published:** 2022-08-04

**Authors:** Marco Rubatto, Martina Merli, Gianluca Avallone, Andrea Agostini, Luca Mastorino, Virginia Caliendo, Amelia Barcellini, Viviana Vitolo, Francesca Valvo, Maria Teresa Fierro, Simone Ribero, Pietro Quaglino

**Affiliations:** ^1^Department of Medical Sciences, Section of Dermatology, University of Turin, Turin, Italy; ^2^National Center of Oncological Hadrontherapy (Fondazione CNAO), Pavia, Italy; ^*^Co-first authors; ^#^Co-first senior authors

**Consent was added to this article:** The patient mentioned in the paper signed the informed consent statement on 13/10/2020.

Original article: Oncotarget. 2021; 12:1116–1121. 1116-1121. https://doi.org/10.18632/oncotarget.27966

